# Risk Factors of New Cerebral Infarctions After Endovascular Treatment for Basilar Artery Stenosis Based on High-Resolution Magnetic Resonance Imaging

**DOI:** 10.3389/fneur.2020.620031

**Published:** 2021-01-20

**Authors:** Jichang Luo, Long Li, Tao Wang, Kun Yang, Yao Feng, Renjie Yang, Yan Ma, Peng Gao, Bin Yang, Liqun Jiao

**Affiliations:** ^1^China International Neuroscience Institute (China-INI), Beijing, China; ^2^Department of Neurosurgery, Xuanwu Hospital, Capital Medical University, Beijing, China; ^3^Department of Evidence-Based Medicine, Xuanwu Hospital, Capital Medical University, Beijing, China; ^4^Department of Interventional Neuroradiology, Xuanwu Hospital, Capital Medical University, Beijing, China

**Keywords:** basilar artery stenosis, new cerebral infarctions, high-resolution magnetic resonance imaging, smoking, plaque burden, remodeling index

## Abstract

**Objective:** The current study aims to analyze the risk factors of new cerebral infarctions in the distribution of basilar artery (BA) detected by diffusion-weighted imaging (DWI) after endovascular treatment in patients with severe BA stenosis.

**Methods:** Data was collected from the electronic medical records of patients with severely atherosclerotic basilar artery stenosis (≥70%) who underwent endovascular treatment. The plaque characteristics, including the plaque distribution, plaque burden, plaque enhancement index, remodeling ratio, and stenosis degree, were evaluated qualitatively and quantitatively using high-resolution magnetic resonance imaging (HR-MRI) and digital subtraction angiography (DSA). The characteristics of the procedure, such as the type of treatment, balloon diameter, balloon length, stent diameter, and stent length, were analyzed.

**Results:** A total of 107 patients with severe basilar artery stenosis (≥70%) who underwent endovascular treatment were enrolled. The study participants included 77 men and 30 women, with an average age of 61.6 ± 8.1 years. The rate of postoperative new cerebral infarctions was 55.1% (59/107), of which 74.6% (44/59) were caused by artery-to-artery embolism, 6.8% (4/59) due to perforator occlusion, and 18.6% (11/59) were caused by a mixed mechanism. Twelve of 59 patients had ischemic events, with nine cases of stroke and three cases of transient ischemic attacks (TIA). The plaque burden in the DWI-positive group was significantly larger than that in the DWI-negative group (3.7% vs. −8.5%, *p* = 0.016). Positive remodeling was more common in the DWI-positive group than in the DWI-negative group (35.6% vs. 16.7%, *p* = 0.028). Smoking was inversely correlated with the rate of new cerebral infarctions (odds ratio, 0.394; 95% confidence interval, 0.167–0.926; *p* = 0.033).

**Conclusion:** The plaque characteristics are not associated with new cerebral infarctions in the distribution of BA, although a large plaque burden and positive remodeling are more likely to appear in patients with new cerebral infarctions after BA stenting, which warrants further studies with a larger sample size. As for smoking, the inverse correlation with new cerebral infarctions in the BA territory needs large-scale prospective randomized controlled trials to verify.

## Introduction

Basilar artery (BA) atherosclerotic stenosis is a common cause of transient ischemic attacks (TIA) and stroke, accounting for approximately 20% of symptomatic ischemic infarctions of the posterior circulation (1). Endovascular therapy is an effective alternative treatment for drug-refractory BA stenosis. However, endovascular treatment for BA atherosclerotic stenosis has a high perioperative complication rate. The Stenting and Aggressive Medical Management for Preventing Recurrent Stroke in Intracranial Stenosis (SAMMPRIS) trial described that the stroke or death rate due to BA atherosclerotic stenosis was as high as 21.6% after BA stenting (2). However, the new cerebral infarction rate detected by diffusion-weighted imaging (DWI) may be higher given that the definition of stroke is the combination of new cerebral infarctions on DWI and clinical symptoms of neurological deficits, as well as some new cerebral infarctions that have no clinical symptoms and are only detected by DWI (3). Indeed, some studies have reported that the prevalence of new cerebral infarctions discovered by DWI is as high as 70% after endovascular treatment (4, 5).

Although most new cerebral infarctions after endovascular treatment are silent without clinical symptoms, the long-term risk of neurological deficits is enormous. Several studies have found that silent infarctions can lead to subcortical cavities, cortical atrophy, and glial cell proliferation, resulting in long-term complications such as cognitive decline, impaired motor coordination, poor visual reactivity, mental disorders, and even a high risk of long-term stroke (6, 7). Indeed, previous studies have reported that silent infarctions increase the risk of recurrent stroke by 2–3-fold, despite strict control of vascular risk factors (8). Therefore, it is crucial to identify the risk of new cerebral infarctions after endovascular treatment.

Previous studies have shown that silent cerebral infarctions are associated with advanced age, hypertension, and diabetes mellitus in the natural course (7, 9). However, few studies have focused on exploring the risks of new cerebral infarctions in the BA territory after endovascular treatment for BA stenosis, in particular, the relationship between plaque characteristics and new cerebral infarctions. High-resolution magnetic resonance imaging (HR-MRI) is an imaging evaluation technology for plaque characteristics that has emerged in recent years. HR-MRI has advantages including high resolution, visualization of the vascular wall structure, and non-invasiveness, and is considered a reliable evaluation method for evaluating plaque characteristics (10). Furthermore, HR-MRI can be used to assess the plaque location, burden, enhancement ratio, size, length, and area, as well as the remodeling index both qualitatively and quantitatively (11–13). In the current study, we retrospectively explored the risk factors of new cerebral infarctions in the BA territory detected by DWI after endovascular treatment for BA stenosis based on HR-MRI in Chinese individuals. We also sought to determine the risk factors associated with patient demographics and the endovascular treatment procedure.

## Materials and Methods

This study was conducted in accordance with the Good Clinical Practice guidelines and the ethical principles of the Declaration of Helsinki. The study was approved by the Ethics Committee of our center, and given the retrospective nature of the study and the fact that data were analyzed anonymously, the study was exempted from obtaining consent from patients. The demographic characteristics, plaque imaging features, and procedure characteristics were examined to analyze the risk of new cerebral infarctions detected by DWI.

### Study Populations and Demographics

We retrospectively enrolled patients with basilar atherosclerotic stenosis who were treated by endovascular therapy between January 1, 2012, and December 31, 2019. The inclusion criteria were as follows: (1) Patients with symptomatic atherosclerotic stenosis of the BA aged from 18 to 80 years, (2) the degree of stenosis of the lesions was more than 70% confirmed by digital subtraction angiography (DSA), (3) HR-MRI was performed before intervention, and (4) the sequence of DWI was performed 72 h before and after the surgery. Some patients were excluded based on the following criteria: (1) Acute BA occlusion treated by endovascular therapy; (2) endovascular therapy for another intracranial and extracranial vessel disease simultaneously; (3) BA stenosis accompanied with moderate-to-severe stenosis of the vertebral artery; (4) non-atherosclerotic cause of BA stenosis, such as Moyamoya disease, vasculitis, or vascular dissection; and (5) preoperative DWI suggested large-area cerebral infarctions (≥1/2).

The demographic data of patients were collected, including age, gender, body mass index (BMI), vascular risk factors (hypertension, diabetes, hyperlipidemia, coronary heart disease [CHD], qualifying events, smoking history, and drinking history), modified Rankin score (mRS) at admission and discharge, and preoperative and postoperative results of DWI. The evaluation of the new infarction was based on a new high signal on DWI and a new low signal on apparent diffusion coefficient (ADC) imaging after 72 h of intervention compared with pre-operative imaging. If a new infarction was detected in the blood supply area of the BA, such as the brainstem, cerebellum, occipital lobe, or thalamus, the patient was classified as DWI positive (DWI+). If the patient had new infarctions in other districts or no new infarction, the patient was classified as DWI negative (DWI–). As for smoking history, non-smokers were defined as not smoking currently or previously, or as smokers.

### Imaging Protocol and Analysis

All eligible patients were examined by a 1.5T or 3.0T magnetic resonance imaging (MRI) system (MAGNETOM Avanto; Siemens Healthineers) with a standard 8-channel head coil to assess the characteristics of BA stenosis. Multi-sequence scans were performed as follows: time-of-flight magnetic resonance angiography (TOF-MRA), fast spin-echo T1-weighted imaging (T1WI-FSE), and T1-weighted enhanced imaging (T1WI + C). The coronal acquisition parameters were as follows: repetition time, 550 ms; echo time, 27 ms; field of view, 200 × 200 cm; layer thickness, 0.6 mm, and echo-train length, 158. The T1WI + C sequence was scanned 5 min after the intravenous injection of gadopentetate meglumine. All images required reconstruction for image analysis from axial, coronal, and sagittal views.

All plaque images were analyzed using CMRtools (Cardiovascular Imaging Solutions Ltd., UK) by two experienced neuroradiologists who were not involved in statistical analyses. The original data with the type of digital imaging and communications in medicine (DICOM) were required for image analysis. The measurements of the vessel area (VA) and lumen area (LA) were performed on cross-sectional T1-weighted BA images at the maximal lumen narrowing (MLN) or reference sites after zooming in 400 times. We used two lines to divide the axial lumen into four quadrants: ventral, dorsal, left, and right (11, 13). The plaque was considered to belong in the quadrant which had the thickest part of the plaque. If the plaque had a large span and the thickest part was between two quadrants, it was defined as being distributed in more than two quadrants. The reference site was selected at the normal segment of proximal or distal stenosis according to the criteria of WASID (Warfarin vs. Aspirin for Symptomatic Intracranial Disease) where the proximal segment was preferred, but the distal vessel was considered when the proximal segment was diseased (14). Wall area (WA) was measured using VA–LA, and the plaque burden was defined as, [(WA _MLN_-WA _reference_)/VA _MLN_] × 100%. The remodeling index (RI) was defined as, [VA _MLN_/VA _reference_]; RI ≥ 1.05 was defined as positive remodeling, RI ≤ 0.95 as negative remodeling, and RI between 0.95 and 1.05 as intermediate remodeling. The contrast enhancement ratio was measured at the slice of the MLN that was normalized by the signal from adjacent gray matter. The enhancement ratio was calculated by, [signal of plaque (post-contrast)/signal of gray matter (post-contrast)]/[signal of plaque (pre-contrast)/signal of gray matter (pre-contrast)] × 100% (15).

### Interventional Procedure and Analysis

The culprit lesions were treated by neurosurgeons who had more than 15 years of experience with endovascular treatment, including primary angioplasty, balloon-mounted stent placement (Apollo), and self-expansion stent placement (Gateway-Wingspan system). The therapeutic strategies were decided by the operators according to the characteristics of the lesion and their own experience; more details are provided in a previous study (16). All patients received preoperative medication with a combination of aspirin (100 mg daily) and clopidogrel (75 mg daily) started 5 d prior to the procedure, or a loading dose of aspirin and clopidogrel (300 mg each) 1 d before the procedure. During the procedure, systematic heparin was administered by intravenous injection at a dose of 2/3 mg per kg of body weight. The standard of procedure was referred to as the protocol of CASSISS (China Angioplasty and Stenting for Symptomatic Intracranial Severe Stenosis) (17). The three types of intervention, primary angioplasty, self-expansion stent (SES), and balloon-mounted stent (BMS), had different procedures. Angiography with a high-pressure contrast agent was followed by balloon pre-expansion during primary angioplasty. As for SES placement, the procedure was similar to that for primary angioplasty except for the self-expansion stent placement after angiography. As for BMS placement, the balloon expanded the stent mostly without the need for pre-expansion.

The parameters of the procedure were collected, including the vessel diameter (VD) at the most stenotic site and reference site, length of the lesion, intervention method, diameter and length of the balloon and/or stent, and pressure (P) before and/or after expansion. The degree of stenosis was defined as [(1-VD _MLN_/VD_reference_) × 100%] in accordance with the standard of WASID (14). We also defined new composite variables to explore the relationship between the lesion and instruments. The diameter ratio was defined as the maximal diameter of the implant divided by the VD _MLN_ and VD _reference_, respectively.

The BA was divided into three segments according to the branch of the anterior inferior cerebellar artery and the superior cerebellar artery: low, middle, and high segments (18).

### Statistical Analysis

Statistical analyses were performed using SPSS 25.0 (IBM). Continuous variables are described as means ± SD and categorical variables as frequency and percentage. Student's *t*-test or Mann–Whitney *U* test was used for the comparison of continuous variables, and the chi-square test was used for categorical variables. Covariates with a univariate *p*-value < 0.10 were subsequently enrolled in multivariate logistic regression analysis. The odds ratio (OR) with 95% confidence interval (CI) was determined using a logistic regression model. *P*-values < 0.05 were considered to indicate statistical significance.

## Results

### Demographic Characteristics

**Figure 1:**
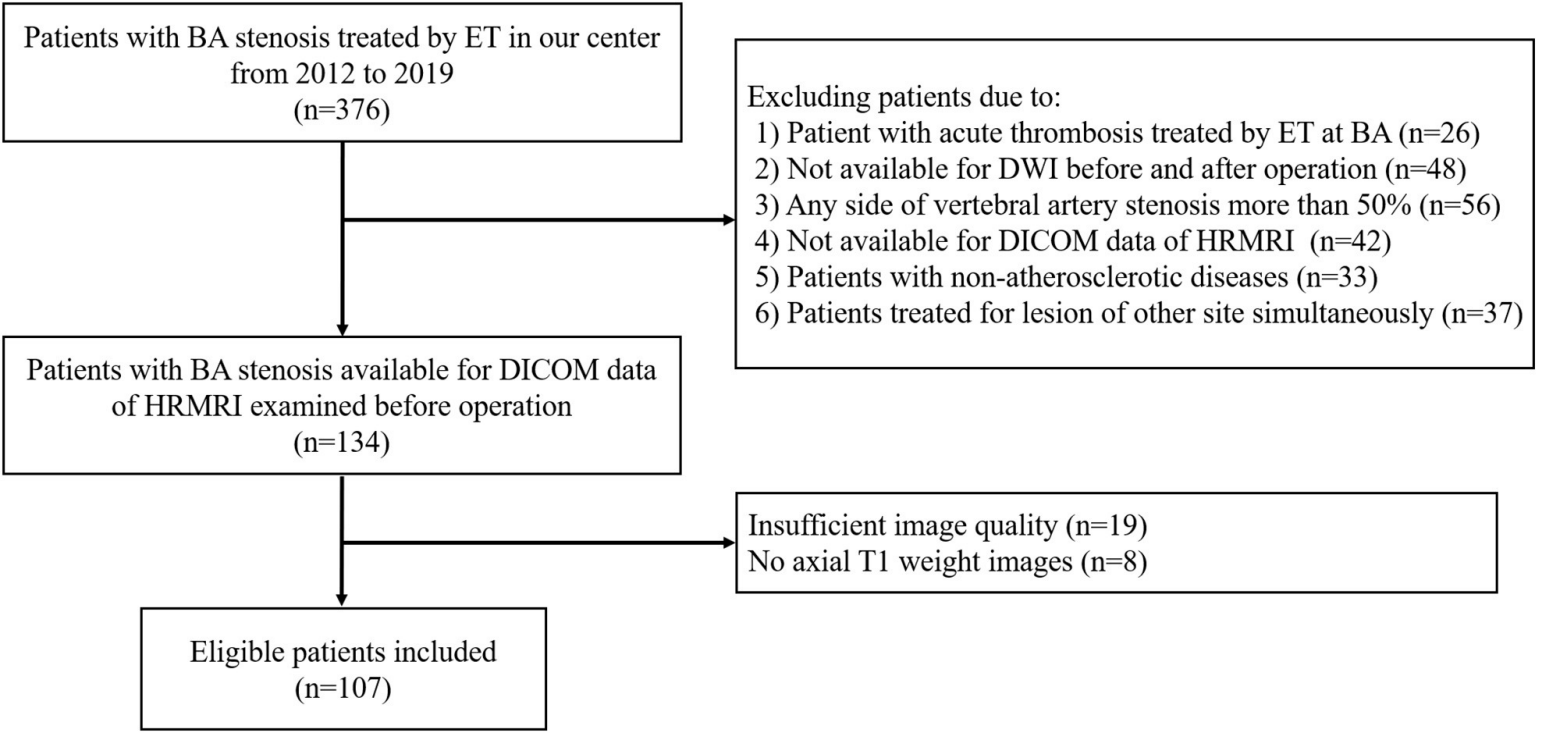
Flow chart.

A total of 376 consecutive patients with BA stenosis underwent endovascular treatment at our center from January 1, 2012, to December 31, 2019; among them, 107 patients were eligible after screening (see Figure 1). The patients comprised 77 men and 30 women with an average age of 61.6 ± 8.1 years. The success rate of endovascular therapy was 100%. The mRs of most patients ranged from 0 to 1, with a proportion of 86.3%. Fifty-nine patients (55.1%) developed new cerebral infarctions after endovascular therapy as evaluated by DWI; among these, 12 patients had ischemic events, with nine cases of stroke and three cases of TIA. There was no death at discharge. As for the mechanisms of infarctions, 74.6% of infarctions (44/59) were caused by artery-to-artery embolism, 6.8% (4/59) by perforator occlusion, and 18.6% (11/59) by a mixed mechanism. There was a significant difference in smoking history (*p* = 0.021) between DWI– and DWI+ patients. The detailed demographic characteristics are described in Table 1.

**Table 1:** Demographic Characteristics.

**Variables**	**All patients** **(*n* = 107)**	**DWI–** **(*n* = 48)**	**DWI+** **(*n* = 59)**	***P*-value**
Age (y)	61.6 ± 8.1	60.7 ± 8.3	62.4 ± 7.9	0.296
Male	77 (72.0%)	37 (77.1%)	40 (67.8%)	0.287
BMI	26.4 ± 3.0	26.3 ± 3.0	26.4 ± 3.0	0.875
Hypertension	88 (82.2%)	39 (81.3%)	49 (83.1%)	0.808
Diabetes Mellitus	46 (43.0%)	18 (37.5%)	28 (47.5%)	0.301
Hyperlipidemia	19 (17.8%)	9 (18.8%)	10 (16.9%)	0.808
CHD	13 (12.1%)	3 (6.3%)	10 (16.9%)	0.092
Arrhythmia	4 (3.7%)	2 (4.2%)	2 (3.4%)	0.609
Smoking history	47 (43.9%)	27 (56.3%)	20 (33.9%)	0.021
Drinking history	29 (27.1%)	13 (27.1%)	16 (27.1%)	0.997
Qualifying event				0.487
TIA	13 (12.1%)	7 (14.6%)	6 (10.2%)	
Stroke	94 (87.9%)	41 (85.4%)	53 (89.8%)	
Pre-operation mRs				0.059
<2	93 (86.9%)	45 (93.8%)	48 (81.4%)	
≥2	14 (13.1%)	3 (6.2%)	11 (18.6%)	

*BMI, body mass index; CHD, coronary heart disease; mRS, modified Rankin Scale*.

### Characteristics Based on HRMRI, DSA, and the Procedure of Endovascular Treatment

**Figure 2:**
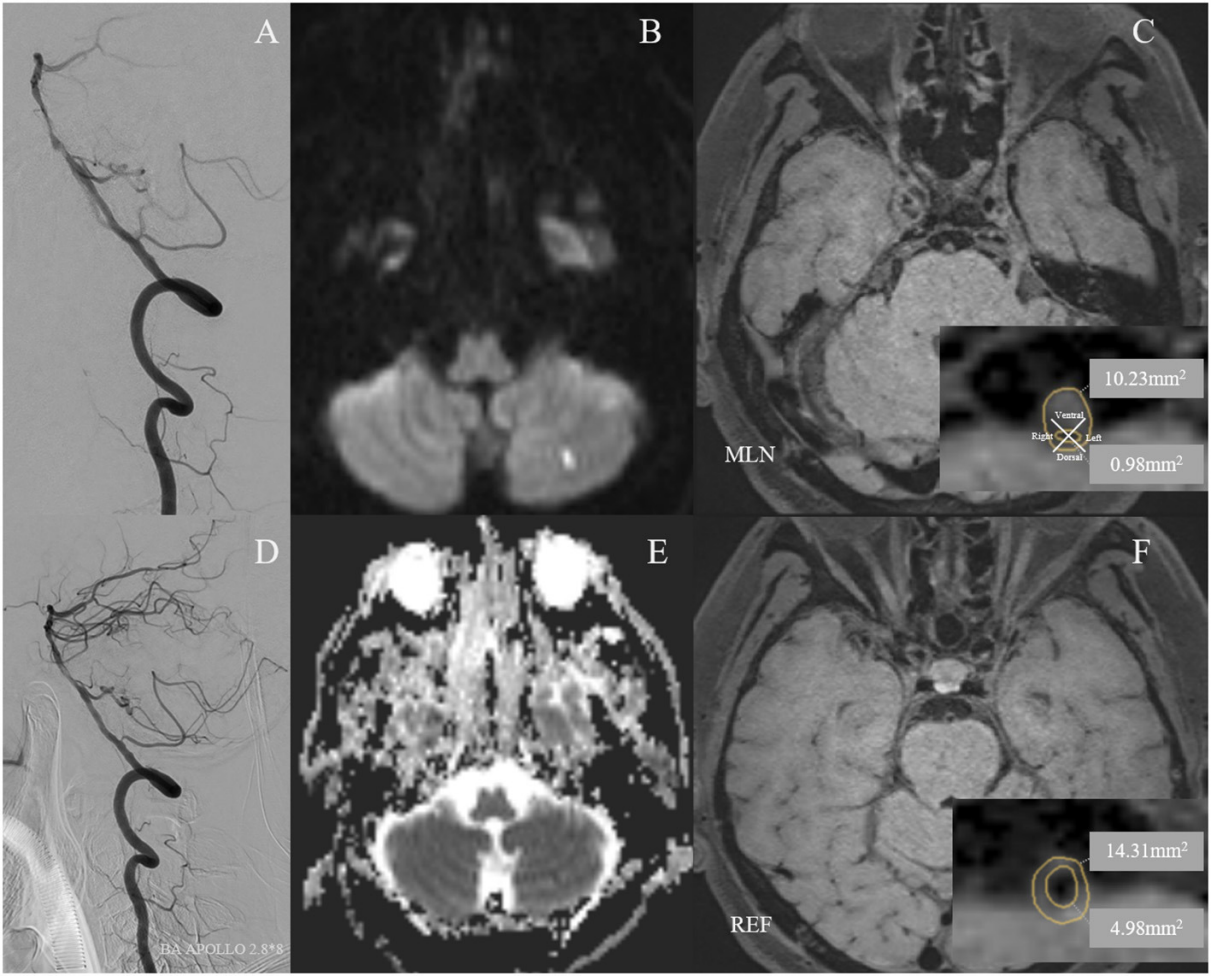
A 59-year-old man with cigarette smoking history and drinking history presented with recurrent transient attacks of dizziness for 2 months. **(A)** Pre-operative digital subtraction angiography (DSA) showed stenosis at the middle segment of the basilar artery (BA) with 75.5% of degree. **(D)** A 2.8 × 8 mm Apollo balloon-mounted stent (MicroPort NeuroTech, Shanghai, China) was placed at the stenotic segment. The patient had a new cerebral infarction at the left cerebellum within 72 h after stenting without clinical symptoms, which was detected by diffusion-weighted imaging with a new high signal **(B)** and apparent diffusion coefficient imaging with a new low signal **(E)**. Figures of the right column are cross-sectional T1-weighted BA images at the maximal lumen narrowing (MLN) **(C)** and reference (REF) **(F)** sites. The plaque was eccentric and belonged to the ventral side of the BA **(C)**. Vessel area (VA) and lumen area (LA) at the MLN (**C**, VA 10.23 mm^2^, LA 0.98 mm^2^) or REF (**F**, VA 14.31 mm^2^, LA 4.98 mm^2^) sites were manually traced for measuring after zooming in 400 times. Wall area (WA) at the MLN or REF sites was calculated by VA-LA. The plaque burden was calculated as [(WA _MLN_-WA _REF_)/VA _MLN_] × 100% and the remodeling index was calculated as VA _MLN_/VA _REF_. Therefore, the plaque burden was −0.78%. The remodeling index of the vessel at MLN was 0.71, which was categorized as negative remodeling.

**Figure 3:**
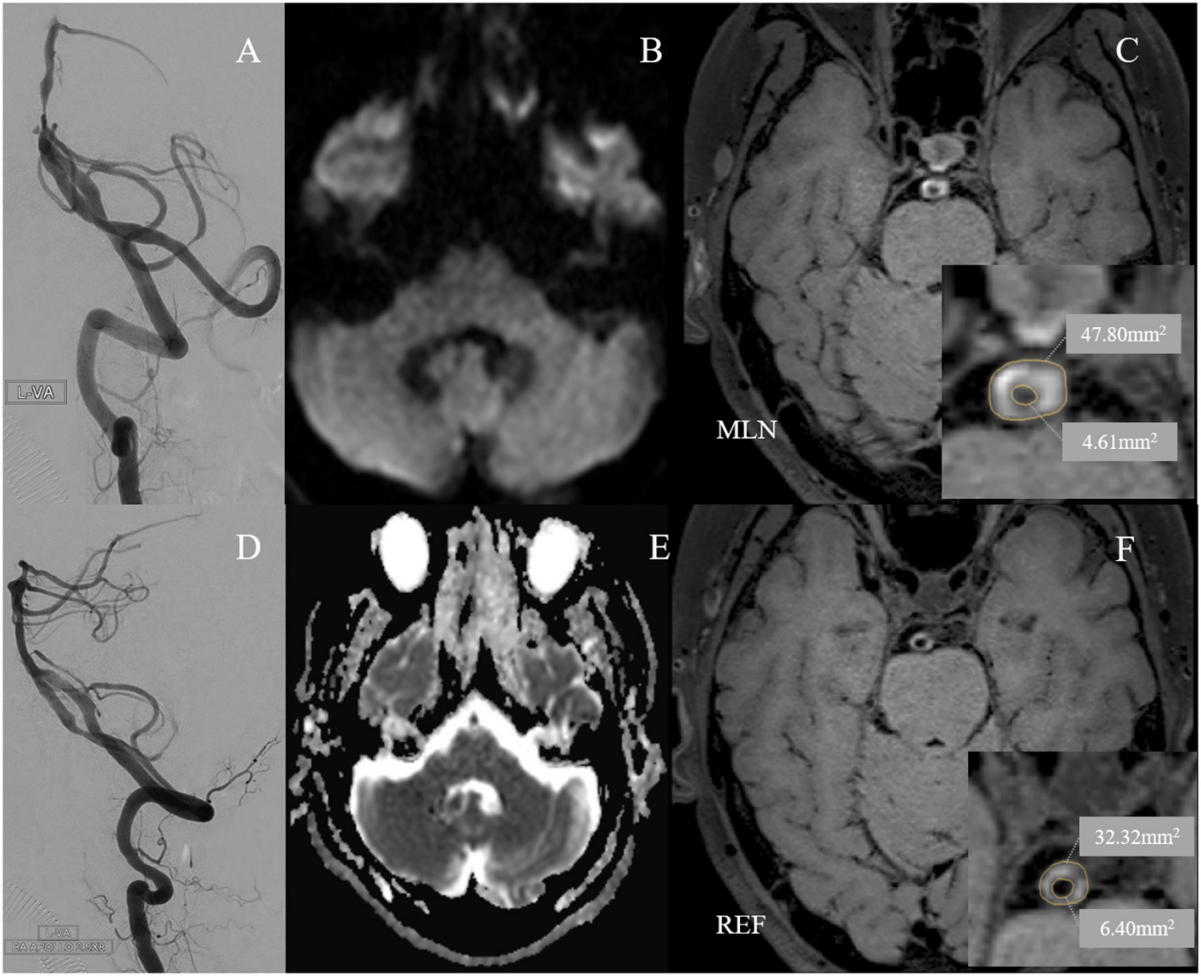
A 60-year-old man with cigarette smoking history presented with recurrent transient attacks of vertigo for 1 month. **(A)** Pre-operative digital subtraction angiography (DSA) showed stenosis at the low segment of the basilar artery (BA) with 77.7% of degree. **(D)** A 2.5 × 8 mm Apollo balloon-mounted stent (MicroPort NeuroTech, Shanghai, China) was placed at the stenotic segment. There was no new cerebral infarction confirmed by diffusion-weighted imaging **(B)** and apparent diffusion coefficient imaging **(E)** within 72 h after stenting. Figures of the right column are cross-sectional T1-weighted BA images at the maximal lumen narrowing (MLN) **(C)** and reference (REF) **(F)** sites. The plaque was concentric and belonged to the circular type **(C)**. Vessel area (VA) and lumen area (LA) at the MLN (**C**, VA 47.80 mm^2^, LA 4.61 mm^2^) or REF (**F**, VA 32.32 mm^2^, LA 6.40 mm^2^) sites were manually traced for measuring after zooming in 400 times. Wall area (WA) at the MLN or REF sites was calculated by VA-LA. The plaque burden was calculated as [(WA _MLN_-WA _REF_)/VA _MLN_] × 100% and the remodeling index was calculated as VA _MLN_/VA _REF_. Therefore, the plaque burden was 36.1%. The remodeling index of the vessel at MLN was 1.48, which was categorized as positive remodeling.

The average stenosis of patients was 77.8 ± 6.8%, with 57.9% of lesions presented at the middle segment of BA from the coronal view and 34.6% at the ventral BA from the axial view. Half of the lesions were eccentric, while the rest presented as either C type or circular type. Most lesions had non-positive remodeling, with a proportion of 72.9%. More than half of the BA stenosis (57.9%) was treated with self-expansion stenosis. The most common site of the lesions was the middle segment of the BA (57.9%) and the ventral region of the BA (34.6%) (Figures 2, 3). There were significant differences in wall area of the reference vessel (17.1 mm^2^ vs. 15.1 mm^2^, *p* = 0.040), plaque burden (−8.5% vs. 3.7%, *p* = 0.016), and positive remodeling (16.7% vs. 35.6%, *p* = 0.028) between DWI– and DWI+ patients. More details are presented in Tables 2, 3.

**Table 2:** Lesion characteristics based on HRMRI and DSA.

**Variables**	**All patients** **(*n* = 107)**	**DWI–** **(*n* = 48)**	**DWI+** **(*n* = 59)**	***P*-value**
Stenosis site (coronal view)				0.506
Low	40 (37.4%)	19 (39.6%)	21 (35.6%)	
Middle	62 (57.9%)	28 (58.3%)	34 (57.6%)	
Low & Middle	5 (4.7%)	1 (2.1%)	4 (6.8%)	
Stenosis site (axial view)				0.141
Ventral	37 (34.6%)	11 (22.9%)	26 (44.1%)	
Side	31 (29.0%)	16 (33.3%)	15 (25.4%)	
Dorsal	18 (16.8%)	9 (18.8%)	9 (15.5%)	
≥2 quadrant	21 (19.6%)	12 (25.0%)	9 (15.3%)	
Plaque morphology				0.245
Eccentric	58 (54.2%)	29 (60.4%)	29 (49.2%)	
C type and circular	49 (45.8%)	19 (39.6%)	30 (50.8%)	
Tandem lesion	8 (7.5%)	2 (4.2%)	6 (10.2%)	0.240
Stenosis (%)	77.8 ± 6.8%	77.8 ± 6.2%	77.8 ± 7.5%	0.961
Diameter at MLN (mm)	0.6 ± 0.2	0.6 ± 0.2	0.6 ± 0.2	0.723
Diameter at reference (mm)	2.8 ± 0.6	2.7 ± 0.6	2.7 ± 0.5	0.565
Plaque length (mm)	6.6 ± 3.2	6.2 ± 2.8	6.9 ± 3.5	0.369
Plaque length/Degree of stenosis	8.5 ± 3.8	8.0 ± 3.5	8.9 ± 4.1	0.273
VA at MLN (mm^2^)	18.6 ± 7.6	18.4 ± 7.9	18.5 ± 7.1	0.666
LA at MLN (mm^2^)	1.2 ± 0.7	1.2 ± 0.8	1.2 ± 0.7	0.757
WA at MLN (mm^2^)	17.4 ± 7.4	17.2 ± 7.8	17.3 ± 6.8	0.539
VA at reference (mm^2^)	21.9 ± 7.0	23.2 ± 7.3	20.8 ± 6.7	0.076
LA at reference (mm^2^)	5.9 ± 2.6	6.1 ± 2.6	5.7 ± 2.6	0.324
WA at reference (mm^2^)	16.0 ± 5.0	17.1 ± 5.4	15.1 ± 4.5	0.040
Plaque burden (%)	−1.8 ± 40.1	−8.5 ± 33.2	3.7 ± 44.4	0.016
Remodeling type				0.028
Positive remodeling	29 (27.1%)	8 (16.7%)	21 (35.6%)	
Non-positive remodeling	78 (72.9%)	40 (83.3%)	38 (64.4%)	
Enhancement ratio	1.7 ± 0.6	1.7 ± 0.6	1.8 ± 0.7	0.965

*MLN, maximal lumen narrowing; VA, vessel area; LA, lumen area; WA, wall area*.

**Table 3:** Characteristics of procedure.

**Variables**	**Total** **(*n* = 107)**	**DWI–** **(*n* = 48)**	**DWI+** **(*n* = 59)**	***P*-value**
Treatment type				
PA	21 (19.6%)	10 (20.8%)	11 (18.6%)	0.943
BMS	24 (22.4%)	11 (22.9%)	13 (22.0%)	
SES	62 (57.9%)	27 (56.3%)	35 (59.3%)	
Diameter _max_ of PTAS (mm)	3.1 ± 0.8	3.0 ± 0.8	3.1 ± 0.8	0.641
Length _max_ of PTAS (mm)	13.6 ± 4.2	13.5 ± 4.3	13.7 ± 4.2	0.832
Pressure _max_ of PTAS (atm)	6.4 ± 1.5	6.4 ± 1.4	6.4 ± 1.6	0.869
Diameter ratio of stenosis^*^	5.8 ± 2.9	5.5 ± 2.5	6.0 ± 3.2	0.446
Diameter ratio of reference^*^	1.2 ± 0.3	1.0 ± 0.3	1.2 ± 0.3	0.441

PA, primary angioplasty; BMS, balloon-mounted stent; SES, self-expansion stent; PTAS, percutaneous transluminal angioplasty with or without stenting.

* *The diameter ratio was defined as the maximal diameter of implant divided by vessel diameter of stenosis and reference*.

### Multivariate Analysis

According to the results of conventional statistical analysis with *p* < 0.10, these covariates, including CHD, smoking history, preoperative mRs, plaque burden, and remodeling index, were included in the multivariate logistic regression analysis. Although the *p*-values of the vessel area and wall area at the reference site were <0.10, they were not included in the multivariate analysis because the plaque burden and remodeling type were calculated. Multivariate analysis demonstrated that smoking was an independent factor for new cerebral infarctions (OR, 0.394; 95% CI, 0.167–0.926, *p* = 0.033). More details are presented in Table 4.

**Table 4:** Risks for post-operative new cerebral infarction assessed by multivariate analysis.

	**OR (95%CI)**	***P*-value**
Age	1.003 (0.952–1.057)	0.905
Smoking history	0.394 (0.167–0.926)	0.033
CHD	4.109 (0.973–17.354)	0.055
Pre-operative mRs		
<2	–	
≥2	2.710 (0.66–11.120)	0.166
Plaque burden	1.001 (0.988–1.014)	0.988
Remodeling ratio		
Non-positive remodeling	–	–
Positive remodeling	2.807 (0.832–9.472)	0.096

*CHD, coronary heart disease*.

## Discussion

The impact of new cerebral infarctions has been largely ignored in clinical practice, especially those cases without clinical symptoms. Most clinical studies, including multicenter randomized controlled studies, only regarded symptomatic cerebral infarctions (stroke) or TIA as the endpoint, which reduces the public's knowledge of new cerebral infarctions without clinical symptoms, known as silent cerebral infarctions (19, 20). Although some new cerebral infarctions have no symptoms, they still pose as a high-risk factor for stroke in the future (8). In addition, silent cerebral infarctions can cause long-term cognitive impairment, impaired motor coordination, and mental illness (6, 21, 22). Therefore, some experts have suggested that the definition of silent cerebral infarctions should be replaced by covert cerebral infarctions, which are difficult to detect by contemporary evaluation methods (23).

The history of endovascular treatment may rapidly increase the probability of new cerebral infarctions without clinical symptoms. However, few studies have reported the incidence of new cerebral infarction after endovascular treatment for BA stenosis. In the current study, the incidence of new cerebral infarctions in the distribution of BA was 55.1%, with 43.9% of them being silent cerebral infarctions, which is consistent with the rate of 15–70% of new cerebral infarctions after carotid stenting (5, 24). Artery-to-artery embolism was the most common infarction mechanism, similar to the findings of previous studies (25). The results demonstrated that smoking, plaque burden, and positive remodeling may be the influencing factors for new cerebral infarctions. Smoking was independently associated with new cerebral infarctions.

### Smoking Is Inversely Associated With the Risk of New Cerebral Infarctions After Endovascular Treatment

As we all know, smoking is a classic risk factor for cardiovascular diseases. Indeed, some studies have reported that the 10-year risk of death was 2-fold in smokers compared to that in non-smokers; smoking has been shown to be the most important risk factor for premature death (26, 27). The damaging effect of smoking has been attributed to several dysfunctions, including endothelial dysfunction, inflammation, and a state of prothrombotic formation (28, 29).

Unexpectedly, there was a paradoxical phenomenon similar to our finding in a large number of studies. Some studies on fibrinolytic therapy for cardiovascular diseases have shown that smokers are associated with better in-hospital and short-term follow-up outcomes. The results of the multivariate analysis demonstrated that smoking was associated with better short-term outcomes (adjusted OR, 0.80; 95% CI, 0.72–0.90) (30). One of the hypotheses was that thrombosis formation was more common in smokers. Another interpretation was that smokers with acute stroke were younger than non-smokers and had lower rates of complications, such as diabetes mellitus and hypertension. Similarly, in the era of endovascular treatment for cardiovascular diseases, the paradoxical effect of smoking was demonstrated in landmark clinical trials, where the short-term prognosis for smokers with acute cardiovascular diseases after mechanical thrombectomy was better than that for non-smokers (adjusted OR: 0.54, 95% CI: 0.38–0.76) (31, 32). Furthermore, previous studies of a subgroup analysis of the International Carotid Stenting Study (ICSS) demonstrated a reduced incidence of 30-day postoperative major adverse events in smokers compared with non-smokers (adjusted OR, 0.33; 95% CI, 0.13–0.85) (33). Some studies have given some explanations for this phenomenon. Dual antiplatelet therapy, including aspirin and clopidogrel, is an essential preoperative preparation for endovascular treatment with conventional or loading doses. Tobacco smoking could enhance the antiplatelet effects of clopidogrel by affecting the enzyme responsible for converting clopidogrel into its active form, which increases the ability to resist platelet aggregation and reduces the probability of thrombogenesis (34, 35). Meanwhile, some researchers suspected that smoking may create a vascular disease state that is more responsive to clopidogrel, even when the patients have not smoked for several years (36).

### Plaque Characteristics Associated With New Cerebral Infarctions After Endovascular Treatment

Although plaque characteristics were insignificant in the multivariate analysis, we found that large plaque burden and positive remodeling was higher in the DWI+ group than in the DWI– group in accordance with univariate analysis. The greater the plaque burden with a large lipid core, the higher the risk of new cerebral infarctions after endovascular treatment, because the risk of the plaque breaking increases during endovascular treatment with external force (37). Similarly, the remodeling index was associated with the risk of new cerebral infarctions. Most studies have defined a remodeling index >1.05 as positive remodeling with plaque advance outward, and a remodeling index <0.95 as negative remodeling with plaque advance inward. In the current study, more than half of patients (65.4%) were negative remodeling, followed by positive remodeling (27.1%) and intermediate remodeling (8.4%). The plaque burden in positive remodeling was larger than that in negative remodeling. Therefore, the risk of new cerebral infarctions was higher in patients with positive remodeling. Some studies have demonstrated that plaque stability is associated with a small plaque burden and negative remodeling. The unstable plaque increased the risk of plaque breakage during endovascular treatment and the rate of new postoperative infarctions (38–40). Furthermore, previous studies have suggested that positive remodeling is more common in patients with advanced BA stenosis (38). However, we found that negative remodeling was a common condition in patients with symptomatic BA stenosis treated by endovascular therapy. The reason for the difference in results was the diversity of eligible patients enrolled in the study. Non-acute, stable patients with symptomatic severe BA stenosis were admitted to our center for endovascular treatment. Studies have found that the development of atherosclerosis is a process that proceeds from positive remodeling to negative remodeling; negative remodeling is a late remodeling form of plaque, with a relatively stable state (41). Some lipid cores may outflow at the onset of an ischemic event due to the instability of the plaque during positive remodeling (39). Therefore, negative remodeling is more common in patients with severe symptomatic stenosis (namely late disease). Therefore, large plaque burden and positive remodeling may be the influencing factors of new cerebral infarctions in the BA territory, although there were insignificances in the multivariate analysis. The small sample size may be the cause of these results, which warrants verification through further studies with larger sample sizes.

### Limitation

The current study has several limitations. First, the study data were limited due to the retrospective nature of the study. It was difficult to subdivide the smoking state of the patients into currently smoking and previously smoking without more details of smoking, however, it has been found that the devastating effects of tobacco smoking on vessels are lasting and do not subside easily after quitting (31). Several studies have reported better short-term outcomes in smokers, including previously and currently smoking compared with non-smokers (42). Second, the evaluation of plaque characteristics is given increasing attention nowadays. HR-MRI is an effective non-invasive method to assess plaque characteristics. However, it was not easily available for patients in our center at the early stage of the study. Third, serum biochemical tests, such as TNF-α levels and intra-arterial oxidative stress, and white matter damage may be related to new cerebral infarctions (43). However, the availability of these examinations was limited in the current analysis.

## Conclusion

Few studies have investigated the risk factors for new cerebral infarctions in the BA territory after endovascular treatment for BA stenosis in Chinese individuals. The plaque characteristics are not associated with new cerebral infarctions in the distribution of BA, although a large plaque burden and positive remodeling are more likely to appear in patients with new cerebral infarctions after BA stenting, which warrants further studies with larger sample sizes. As for smoking, the inverse correlation with new cerebral infarctions in the BA territory should be verified by large-scale prospective randomized controlled trials.

## Data Availability Statement

The raw data supporting the conclusions of this article will be made available by the authors, without undue reservation.

## Ethics Statement

The studies involving human participants were reviewed and approved by the Ethics Committee of Xuanwu Hospital, Capital Medical University. The patients/participants provided their written informed consent to participate in this study.

## Author Contributions

JL contributed to the preparation of the manuscript and data collection. JL, LL, YF, and RY contributed to the data collection. KY and TW contributed to data analysis and interpretation. LJ, YM, PG, and BY contributed to the experimental design and manuscript revision. All authors contributed to the article and approved the submitted version.

## Conflict of Interest

The authors declare that the research was conducted in the absence of any commercial or financial relationships that could be construed as a potential conflict of interest.
